# Psychometric testing of the theory of planned behavior–based self-employment intention scale and its application among Chinese undergraduate nursing students

**DOI:** 10.1186/s12912-025-04172-9

**Published:** 2025-11-26

**Authors:** Wei Yang, Feng Song, Hai-Yan Jing, Yun-Hang He, Ming-Yue Li, Kun Jing, Jia-Liang Xi, Yu-Lin Lu, Nian-Qi Cui

**Affiliations:** 1 School of Nursing, Kunming Medical University, Kunming, 650500 China; 2 Faculty of Medicine, The National University of Malaysia, Bangi, 43600 Malaysia; 3 Faculty of Medicine, Yunnan College of Business Management, Kunming, 650032 China; 4 Luoyang Orthopaedic Hospital of Henan Province (Henan Provincial Orthopaedic Hospital), Zhengzhou, 450000 China; 5 Cardiovascular Surgery Department, Kunming Yan’an Hospital, Yunnan Provincial Institute of Cardiovascular Surgery, Kunming, 650051 China; 6 Faculty of Health Science and Sports, Macao Polytechnic University, Macao, 999078 China; 7 Department of Nursing, Peking University People’s Hospital, Beijing, 100044 China

**Keywords:** Self-employed nurse, Nursing students, Theory of planned behavior, Reliability, Validity, Cross-sectional study

## Abstract

**Objective:**

The main research objectives include: (1) translating the English version of the Planned Self-Employment Scale (PSES), developed within the Theory of Planned Behavior, into Chinese and assess its reliability and validity among Chinese undergraduate nursing students, thereby establishing the Chinese version of PSES; and (2) employing the Chinese version of PSES to survey self-employment intentions and influencing factors among Chinese undergraduate nursing students.

**Methods:**

In the initial psychometric testing phase, the translation and cross-cultural adaptation of the PSES into the Chinese was conducted according to guideline for Cross-Cultural Adaption. The recruitment of 8 nursing experts to conduct face validity assessments through face-to-face interviews and evaluate both qualitative and quantitative content validity. From January to March 2022, 300 undergraduate nursing students were recruited from the investigator institutions to assess the reliability and validity of the Chinese version of the PSES through a single-center cross-sectional study. The psychometric properties of the PSES were evaluated through item analysis, reliability and validity. Item analysis was conducted using the critical ratio (CR) method and Pearson correlation coefficients. Validity tests included face validity, content validity, construct validity, and convergent validity. Reliability tests included Cronbach’s α coefficient and test-retest reliability. In the survey study phase, convenience sampling was used. 936 undergraduate nursing students were recruited from all 10 institutions which offer baccalaureate nursing programs within the same province from April to July 2022 for a multi-center cross-sectional study using the Chinese version of the PSES. This phase examined self-employment intention and its influencing factors among Chinese undergraduate nursing students.

**Results:**

In the initial psychometric testing phase, the Chinese version of the 12-item scale demonstrated good psychometric properties. Item analysis indicated that no items required removal. The face validity was evaluated by 8 experts, who agreed that the items’ appearance is consistent with the scale’s overall goal. Both quantitative and qualitative content validity evaluations demonstrated that the Chinese version of the PSES maintained excellent content validity. Confirmatory factor analysis showed good fit indices: χ²/df = 3.58, CFI = 0.93, RFI = 0.91, IFI = 0.95, NFI = 0.93, and TLI = 0.93. Reliability measures were also robust, with a total Cronbach’s α coefficient was 0.92, and the test-retest reliability was 0.93. In the survey study phase, the average score of intentions of self-employment of Chinese undergraduate nursing students was 3.60 ± 1.20, which was at a moderate level. Structural equation analysis showed that Attitude (β = 0.14, *P* < 0.001), Subjective Norm (β = 0.19, *P* < 0.001), and Perceived Behaviour Control (β = 0.49, *P* < 0.001) had a significant positive effect on Intention.

**Conclusion:**

The Chinese version of the PSES scale has good reliability and is applicable to nursing students in China. Policy makers should enhance pertinent laws and regulations, increase nursing students’ recognition of self-employed nurses, enhance social support, and cultivate nursing students’ innovative and entrepreneurial abilities to increase self-employment intention.

**Clinical trial number:**

Not applicable.

**Supplementary Information:**

The online version contains supplementary material available at 10.1186/s12912-025-04172-9.

## Introduction

Being self-employed involves acting as your own boss, whether or not you have employees [1]. This concept was first introduced in the context of recognizing the diverse opportunities available to nurses beyond traditional employment settings. At the start of the previous century, self-employed nurses ran their own practices as ‘private duty nurses’ working independently and receiving direct payment from private patients requiring nursing care [2]. Currently, self-employed nurses are defined as registered nurses who operate independently rather than working as employees in healthcare institutions [3]. Although the concept of self-employed nurses has not been formally introduced in China, the emergence of “internet + nursing services” has enabled licensed nurses who have left medical institutions and operate independently to contract with online nursing platforms. These nurses provide home-based nursing services, fulfilling a role similar to that of self-employed nurses [4]. Self-employed nurses play a significant role in promoting community health. On the one hand, they are capable of delivering community-based services that extend to patients’ homes or clinics beyond the hospital setting, such as diabetes education and management, ostomy care, and other specialized interventions [5]. This enables the provision of professional long-term care for individuals with mobility impairments, disabilities, and the elderly, effectively addressing the pressing demands of home-based care [6]. On the other hand, this can effectively contribute to expanding the range of primary health care services available to the population and improve access to health care [7]. In remote areas, they establish clinics to deliver convenient and affordable medical and preventive education, thereby improving community health and economic prospects [8]. Furthermore, our previous research has demonstrated that self-employed nurses can help mitigate the current nursing staff shortage and contain healthcare expenditures [9].

The rapid processes of globalization, social transformations, and labor market pressures have profoundly reshaped the landscape of the nursing employment market and nurses’ career opportunities [10, 11], while intensifying the challenges associated with nurse employment [12]. Simultaneously, systemic neglect of nurse well-being is exacerbating the already severe shortage of healthcare workers. Factors such as poor working conditions, low wages, excessive workloads, and emotional exhaustion collectively endanger nurses’ physical and mental health, propelling a growing number of nurses toward burnout or attrition from the profession [13]. In the International Council of Nurses’ National Nurses’ Association survey, 48.4% of countries reported a moderate to significant rise in the proportion of nurses leaving the industry since 2021 [14]. As early as 2016, over 12.6 million individuals had applied to become self-employed nurses [12].

In this context, self-employed nurses evidently present employment opportunities for new graduates. Although a specialized social security agency for self-employed nurses exists, it currently offers guidance solely on managing relationships between self-employed nurses, their clients, and nursing care services, without establishing specific regulations for new graduates aspiring to enter self-employment [12]. Furthermore, the implementation of self-employed nursing faces multiple barriers, including the absence of standardized entry criteria, delays in licensing, obstacles to securing health insurance reimbursement, and challenges in accessing occupational benefits [9]. As a result, self-employment remains particularly arduous for new graduates. Simultaneously, becoming a self-employed nurse demands comprehensive professional competencies [15]; yet, many new graduates lack the fundamental clinical judgment skills and entrepreneurial capabilities required for safe practice [16, 17]. Consequently, educators should prioritize fostering nursing students’ cognitive and practical competencies in self-employment to meet labor market needs. Thus, understanding nursing students’ intentions to be self-employed nurses and the factors influencing these intentions would be highly advantageous. This would aid healthcare systems and nursing industry leaders in achieving human resource balance [18]. Additionally, it provides valuable insights for universities to adapt curricula and develop targeted training programs. However, China currently lacks both appropriate instruments for measuring self-employment intention and research related to self-employed nurses. Consequently, it remains challenging to understand nursing students’ self-employment intentions and the influencing factors.

Certain theory, such as the Theory of Planned Behavior, may serve as valuable references for investigating the influencing factor of intention. The Theory of Planned Behavior, proposed by Ajzen in 1991 [19], suggests that intention is influenced by three factors: Attitudes, Perceived Behaviour Control, and Subjective Norm. At present, no instrument exists in China to examine the factors affecting nursing students’ intentions to pursue self-employment. Nonetheless, Bulfone et al. [12] developed the Planned Self-Employment Scale (PSES) to assess the nursing students’ intentions to be self-employed nurses and to identify the factors influencing their intentions based on the Theory of Planned Behavior. The English version of the PSES consists of four dimensions (Attitude, Subjective Norm, Perceived Behaviour Control, and Intention) and 12 items, and the scale demonstrates good reliability and validity and has been validated with nursing students in Italy.

Therefore, the main research objectives include: (1) translating the English version of the PSES, developed within the Theory of Planned Behavior, into Chinese and assess its reliability and validity among Chinese undergraduate nursing students, thereby establishing the Chinese version of PSES; and (2) employing the Chinese version of PSES to survey self-employment intentions and influencing factors among Chinese undergraduate nursing students.

## Methods

### Phase 1: cross-cultural adaptation and psychometric evaluation of the Chinese version of the PSES

#### Design and procedure

In this phase, the PSES was translated from English to Chinese following the guidelines for the Cross-Cultural Adaptation [20]. Subsequently, the reliability and validity of the Chinese version of the PSES were assessed through a single-center cross-sectional study.

#### Translation procedure

The translation phases were as follows according to the guidelines for the Cross-Cultural Adaptation [20].

Translation process: (1) Initial translation: Two translators, who scored highly on the International English Language Testing System (IELTS), translated the PSES from English to simplified Chinese. One translator had a background in medical English and was familiar with the scale, whereas the other had no medical background. (2) Synthesis of the translations: After discussion and analysis, the translations from the two translators were combined into a synthesized version. (3) Back translation: Two native English speakers back-translated the Chinese text into English. The back translation version was then compared to the original version, and slight wording changes were made to better fit the Chinese cultural context. (4) Expert committee: The expert committee was consulted regarding the relevance, conceptual equivalence, and clarity of expression for each item in the Chinese version of the PSES. The committee comprised methodologists, language experts, nursing specialists, and translators. Consensus was reached, resulting in a prefinal Chinese version of the PSES that achieved semantic, habitual, and conceptual equivalence. (5) Test of the prefinal version: A group of 38 nursing students, selected through convenience sampling, voluntarily completed the pretest of the Chinese version of the PSES. Afterward, they were encouraged to provide feedback and suggestions for revisions. The pretest results demonstrated that the final Chinese version of the PSES exhibited good readability and comprehension. (6) Submission of documentation to the developers for appraisal of the adaptation process: The documents were submitted to the scale developers for validation and further guidance. On the basis of their feedback, no revisions were made, and the final Chinese version of the PSES was confirmed, comprising 12 items across 4 dimensions.

#### Measurements

In this phase, we translated the English version of the Planned Self-Employment Scale and assessed its reliability and validity to develop the Chinese version of the PSES. The English version of the PSES was developed by Bulfone et al. in 2020 [12], comprising four dimensions, each containing three items, resulting in a total of 12 items. These dimensions are attitude, subjective norm, perceived behaviour control, and intention. The scale uses a 7-point Likert scale, with responses ranging from ‘totally disagree = 1’ to ‘totally agree = 7.’ The total score ranges from 12 to 84, while the scores for each dimension range from 3 to 21. Higher scores on each dimension indicate a stronger self-employment intention, more positive attitudes, and greater perceived behaviour control, while lower scores on the subjective norm dimension reflect less social pressure regarding self-employment.

#### Setting

In January 2022, 8 nursing experts were recruited from a university in Yunnan Province that offers baccalaureate nursing programs to conduct face validity assessments and to evaluate the content validity both qualitatively and quantitatively. Undergraduate nursing students were recruited from the same university, from January to March 2022, and the reliability and validity of the Chinese version of the PSES were assessed in a single-center cross-sectional study.

#### Participants

For face and content validity assessment, it is recommended that the number of experts be at least six but not exceeding ten; therefore, the recruitment of 8 nursing experts to conduct face validity assessments through face-to-face interviews and evaluate both qualitative and quantitative content validity was deemed appropriate [21]. In this phase, which involved a single-center cross-sectional survey, the sample size was determined based on the guideline that it should be 5 to 10 times the number of items on the scale [22]. The Chinese version of the PSES comprises 12 items, and accounting for a potential 20% invalidity rate of questionnaires, the minimum required sample size was calculated as 72 participants [22]. Furthermore, factor analysis recommendations suggest a sample size between 100 and 400 [23]. Integrating both criteria, this study utilized convenience sampling to recruit 300 undergraduate nursing students for the reliability and validity testing of the Chinese version of the PSES, a sample size considered appropriate.

The inclusion criteria were as follows: (1) undergraduate nursing students enrolled in baccalaureate nursing programs and (2) choosing to take part in the survey voluntarily. The following were the exclusion criteria: (1) the time to fill out the questionnaire was estimated to be less than 5 min and (2) the answers to the questionnaires filled out had obvious regularity.

#### Data collection

This stage involved the recruitment of undergraduate nursing students at the researcher’s institution using convenience sampling. The researcher contacted the dean of the institution’s school of nursing and distributed the link of the questionnaire to the WeChat groups.

Data collection of the survey was conducted using the Sojump platform (https://www.wjx.cn/vm/elr4EcA.aspx) to ensure that respondents only submitted the questionnaire after completing all the questions without missing data. The survey included information about the purpose of the study, its content and procedures for completion. In addition, a minimum time limit of five minutes was set for completing the questionnaire to maintain the reliability of the data collected and increase the accuracy of the survey results. Only one survey could be completed per IP address and mobile device. The online survey platform utilized in this study was configured to mandate responses for all items, thereby precluding incomplete data and eliminating the necessity for multiple imputation methods. Before the analysis, questionnaires where all responses were identical or completed in under 5 min were deemed invalid and excluded.

#### Data analysis

The statistical analyses were conducted with SPSS 24.0 (IBM, Chicago, IL, United States), AMOS 24.0 (Amos Development Corporation, Chicago, IL, United States) for item analysis, reliability and validity testing. Item analysis was conducted using the critical ratio (CR) method and Pearson correlation coefficients. Reliability tests included Cronbach’s α coefficient and test-retest reliability. Cronbach’s α coefficient were used to assess internal consistency reliability, while test-retest reliability was used to evaluate stability. Validity tests included face validity, content validity, construct validity, and convergent validity. The level of significance was established at α = 0.05.

**(1) Item analysis**

Item analysis included critical ratio and correlation analysis. (a) Critical ratio: In the CR method, participants’ total scale scores were ranked in descending order, with the top 27% and bottom 27% being selected as the high and low-score groups, respectively. Independent samples t-tests were performed to compare the scores of items between the two groups. Items with a CR value less than 3.00 or without a statistically significant difference between the groups (*P* >0.05) were considered for removal [24]. (b) Correlation analysis: Pearson correlation analysis was employed to assess the item-total consistency of the questionnaire. Items with a correlation coefficient r below 0.20 or a P value greater than 0.01 were eliminated [24].

**(2) Validity test**

- Face validity: 8 nursing experts participated in face-to-face interviews to evaluate the Chinese version of the PSES’s comprehensibility, relevance, and clarity. Face validity was assessed according to expert viewpoint.
- Content validity: For the qualitative assessment, an interview guide was developed addressing grammatical structure, appropriate wording, item formulation, and scaling to conduct face-to-face interviews with 8 nursing experts. The Chinese version of the PSES was modified based on their feedback. For the quantitative assessment, the Item-level content validity index (I-CVI) and Scale-level Content Validity Index (S-CVI/Ave) were employed to evaluate content validity [25]. Each item’s relevance in the Chinese version of the PSES was evaluated using a 4-point Likert scale, with the dimension of the scale on a 4-point scale (1 = not relevant, 2 = weakly relevant, 3 = relatively relevant, 4 = highly relevant).
- Construct validity: Exploratory factor analysis (EFA) and confirmatory factor analysis (CFA) are commonly used to evaluate structural validity. Earlier research [26] has indicated that EFA and CFA serve different purposes, with EFA being used during the scale development phase and CFA being applied in the validation phase. For culturally adapted scales, CFA alone is generally sufficient, and model modifications are made on the basis of fit indices [26]. If the modified model is unacceptable, EFA should be reconducted [26]. An ideal value for the χ2/df ratio is < 3, and values < 5 are acceptable [27]. Fit indices such as the goodness of fit index (GFI), incremental fit index (IFI), comparative fit index (CFI), normed fit index (NFI), tucker-lewis index (TLI), and adjusted goodness-of-fit index (AGFI) need to be greater than 0.8 [28].
- Convergent validity: Convergent validity is the extent to which different measures yield similar results when assessing the same objective. Indicators often employed to assess convergent validity included average variance extracted (AVE) and construct reliability (CR). When the AVE is above 0.50 and the CR is over 0.70, convergent validity is deemed satisfactory [29].

**(3) Reliability test**

- Internal consistency: Cronbach’s α coefficient was used to evaluate the reliability of internal consistency. A Cronbach’s α coefficient of 0.7 or higher was considered acceptable for reliability [30].
- Test-retest reliability: Earlier studies indicate that having 30 to 50 participants is generally adequate for evaluating test-retest reliability [31, 32]. As a result, this research chose a group of 30 students for the second assessment, carried out after a two-week gap. The scale was considered to have stable reliability if the correlation coefficient between the two assessments was greater than 0.70 [33].

### Phase 2: intentions and influencing factors of self-employment among Chinese undergraduate nursing students

#### Design and procedure

During the second phase, researchers conducted a multi-center cross-sectional study to explore intentions and influencing factors of self-employment among Chinese undergraduate nursing students, which utilized the Chinese version of the PSES.

#### Measurements

In this phase, a multicenter cross-sectional survey was conducted utilizing the Chinese version of the PSES to assess the level of self-employment intention and its influencing factors among Chinese undergraduate nursing students. The survey questionnaire consisted of the Chinese version of the PSES and a general information questionnaire. The Chinese version of the PSES, which contains 12 items, demonstrated good psychometric properties. The general information questionnaire was a self-developed tool designed to collect demographic details such as age, gender, ethnicity, average monthly household income per capita, grade, et al.

#### Setting

Undergraduate nursing students were recruited from 10 institutions which are all undergraduate colleges and universities offering baccalaureate nursing programs within the same province from April to July 2022 for a multi-center cross-sectional study.

#### Participants

In the second phase, a multi-center cross-sectional survey was conducted, the sample size was determined based on the guideline that it should be 5 to 10 times the number of items on the scale [22]. The Chinese version of the PSES comprises 12 items, and accounting for a potential 20% invalidity rate of questionnaires, the minimum required sample size was calculated as 72 participants [22]. Furthermore, structural equation modeling requires a minimum sample size of 200 [34]. Given our objective to comprehensively reflect the current state of self-employment intention among undergraduate nursing students, we recruited participants from all undergraduate colleges and universities offering baccalaureate nursing programs in Yunnan Province. Consequently, rather than adhering to the minimum sample size requirement, we opted for the maximum sample size attainable through convenience sampling. 1,361 questionnaires were gathered, with 936 deemed suitable for analysis, and an effective response rate of 68.77%.

The eligibility criteria were identical to those used in the first stage.

#### Data collection

Carrying out a survey in the second phase, the researcher contacted the chairman of the Nursing Education Committee of the Yunnan Nursing Association, who then contacted the deans of the school of nursing of all undergraduate colleges and universities in Yunnan Province and asked the deans to distribute the link of the questionnaire in the WeChat groups. The data collected was checked for validity by the researcher.

In the second stage, the method for collecting data was identical to that used in the first stage.

#### Data analysis

Partial least squares structural equation modeling analysis (PLS-SEM) was performed using Smart PLS 3.0 (SmartPLS GmbH, D-22114 Oststeinbek, Germany).

Structural equation modeling (SEM) is used to explore influencing factors of self-employment among Chinese undergraduate nursing students in this study. The model’s fit was tested using a path coefficient (R²) for each endogenous latent variable [35]. The higher the R² value, the stronger the explanatory ability of the model and the better the fit. A model with an R² value over 0.67 had strong explanatory power, while an R² near 0.33 indicated moderate power, and an R² under 0.19 showed weak power [36].

#### Ethical considerations and consent to participate

Original author of the English Version of the PSES granted approval and authorization to the researchers for the adaptation of the original version of PSES into Chinese. The study was approved by the ethics committee of the Yunnan College of Business Management (No. 2021007). Participants were told that their participation was both voluntary and anonymous, and that all questionnaire data would be used solely for study analysis and kept confidential. All participants provided verbal informed consent and could quit the study anytime.

## Results

### Cross-cultural adaptation and psychometric evaluation of the Chinese version of the PSES

#### Participant characteristics

8 experts involved in assessing content and face validity comprised 4 nursing education specialists, 3 nursing management specialists, and 1 clinical nursing specialist. Among them, 2 held doctoral degrees, 5 possessed master’s degrees, and 1 had a bachelor’s degree. Additionally, 7 experts held associate senior professional titles or higher. The mean age of the panel was 51.38 years, with professional experience distributed as follows: 1 expert had 5–10 years, 3 had 10–20 years, and 4 had over 30 years of experience.

There was a balanced gender ratio among the participants according to their demographic characteristics, with a slightly greater proportion of females (54.00%). The age of undergraduate nursing students ranged from 18 to 25 years. Han Chinese participants accounted for 65.67% of the sample, and 40.67% of the participants reported a monthly household income between 2,001 and 4,000 RMB. The contents are summarized in Table 1.

**Table 1:** Demographic details of the study participants (*N* = 300)

Variables		*N* (%)/M ± SD
Gender	Men	138 (46.00)
	Women	162 (54.00)
Age (years)	18–20	197 (65.67)
	21–25	103 (34.33)
		20.20 ± 1.40
Ethnicity	Han Chinese	197 (65.67)
	Minority nationality	103 (34.33)
Average monthly household income per capita	≤ 2000¥ /278.20$	101 (33.67)
	2001 ~ 4000¥ /278.34$–553.40$	122 (40.67)
	4001 ~ 6000¥ /556.54$–834.60$	52 (17.33)
	6001 ~ 8000¥ /834.74$–1112.80$	14 (4.67)
	> 8000¥ />1112.80$	11 (3.67)
Grade	Freshman	114 (38.00)
	Sophomore	71 (23.67)
	Junior	45 (15.00)
	Senior	70 (23.33)
Father’s education level	Primary or low	111(37.00)
	Middle high school	129 (43.00)
	High or vocational school	40 (13.33)
	Undergraduate and above	20 (6.67)
Mother’s education level	Primary or low	154 (51.33)
	Middle high school	98 (32.67)
	High or vocational school	27 (9.00)
	Undergraduate and above	21 (7.00)
The preferred monthly remuneration	2000 ~ 4000 ¥/278.20$–553.40$	24 (8.00)
	4001 ~ 6000 ¥/556.54$–834.60$	66 (22.00)
	6001 ~ 8000 ¥/834.74$–1112.80$	82 (27.33)
	8001 ~ 10,000 ¥/1112.80$–1391.00$	47 (15.67)
	> 10,000 ¥/>1391.00$	81 (27.00)

#### Item analysis

All items in the Chinese version of the PSES reached statistical significance (*P* < 0.001). For the Chinese version of the PSES, the Pearson correlation coefficients between the 12 items and the total score ranged from 0.61 to 0.82 (*P* < 0.001). No correlation coefficients were found to be less than 0.40; therefore, no items were removed. The contents are summarized in Table 2.

**Table 2:** Item analysis of the Chinese version of the PSES (*N* = 300)

Item	Pearson Correlation	*P*	Extreme group comparison	*P*
	Item-total correlation		Critical ratio (CR)	
A1	0.61	<0.001	10.08	<0.001
A2	0.69	<0.001	11.67	<0.001
A3	0.75	<0.001	14.83	<0.001
SN4	0.75	<0.001	14.49	<0.001
SN5	0.78	<0.001	14.90	<0.001
SN6	0.79	<0.001	14.39	<0.001
PBC7	0.64	<0.001	12.05	<0.001
PBC8	0.78	<0.001	17.03	<0.001
PBC9	0.62	<0.001	12.53	<0.001
I10	0.82	<0.001	20.92	<0.001
I11	0.78	<0.001	18.05	<0.001
I12	0.79	<0.001	18.54	<0.001

#### Validity test

**Fig. 1:**
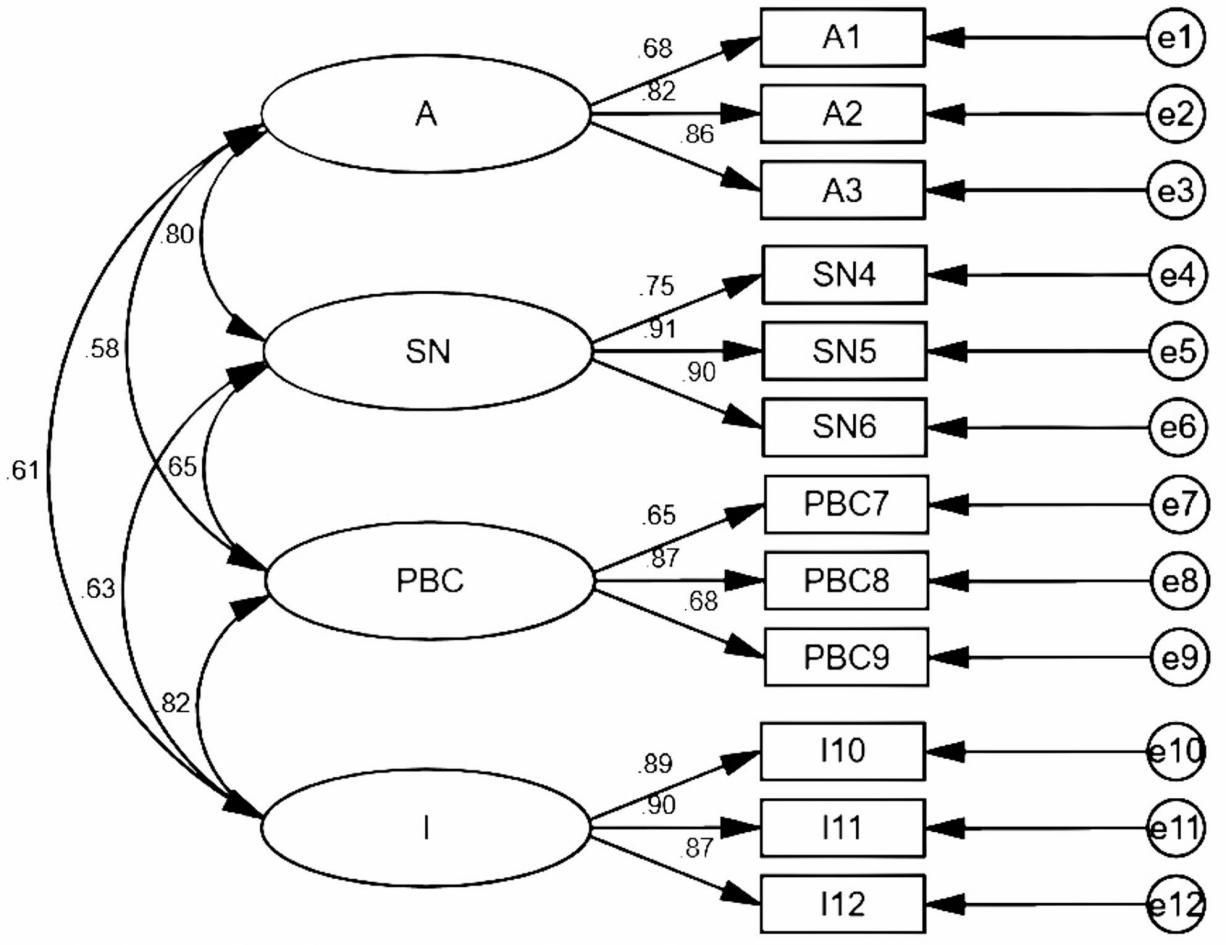
Modified results of the confirmatory factor analysis of the Chinese version of the PSES

- Face validity: 8 experts evaluated face validity, agreeing that the items’ appearance aligns with the scale’s overall purpose. The interview guide can be found in Supplementary File 2.
- Content validity: A survey of 8 experts was conducted, and their recommendations were used to enhance the scale’s content quality. The interview guide can be found in Supplementary File 2. In the Chinese version of the PSES, the I-CVI values for each item varied between 0.88 and 1.00, with the overall scale’s S-CVI/Ave being 0.99. These outcomes demonstrate that the Chinese version of the PSES maintains excellent content validity.
- Construct validity: All fit indices met standard research criteria, suggesting the model exhibited good overall fit. Consequently, EFA was not performed. The contents are summarized in Table 3; Fig. 1.
- Convergent validity: For the four dimensions of the Chinese version of the PSES, the AVE values were 0.63, 0.73, 0.54, and 0.79, and the CR values were 0.83, 0.89, 0.78, and 0.92, respectively. Except for the CR value of the third dimension, which was slightly below the ideal threshold of 0.80, all the other values were acceptable, suggesting that the Chinese version of the PSES demonstrates strong convergent validity.

**Table 3:** The Chinese version of the PSES confirmatory factor analysis (*N* = 300)

Index	χ²/df	RMSEA	RFI	IFI	NFI	TLI	CFI
Recommended parameter	3–5	< 0.10	> 0.90	> 0.90	> 0.80	> 0.90	> 0.90
Value	3.58	0.09	0.91	0.95	0.93	0.93	0.95

#### Reliability test

- Internal consistency: The internal consistency estimates for the four dimensions of the PSES ranged from 0.77 to 0.92, with a Cronbach’s α coefficient of 0.92. The contents are summarized in Table 4.
- Test‒retest reliability: The scale demonstrated an overall test‒retest reliability of 0.93, which implies acceptable reliability.

**Table 4:** The dependability of the scale and its individual dimensions (*N* = 300)

Dimensions	Number of items	Cronbach’s α coefficient
Attitude (A)	3	0.83
Subjective Norm (SN)	3	0.88
Perceived Behaviour Control (PBC)	3	0.77
Intention (I)	3	0.92
Total scale	12	0.92

### Intentions and influencing factors of self-employment among Chinese undergraduate nursing students

#### Demographics of the participants

The age of undergraduate nursing students ranged from 18 to 25 years. Of the participants, 87.93% were female, most students were ethnic Han. The contents are summarized in Table 5.

**Table 5:** Demographic details and the differences on the intentions of participants (*N* = 936)

Variables		*N* (%)	M ± SD	*P*
Gender	Men	113 (12.07)	3.50 ± 1.30	0.693
	Women	823 (87.93)	3.60 ± 1.20	
Age (years)	18–20	291 (31.10)	3.50 ± 1.20	0.031
	21–25	645 (68.91)	3.60 ± 1.20	
Ethnicity	Han Chinese	679 (72.54)	3.50 ± 1.20	0.168
	Minority nationality	257 (27.46)	3.70 ± 1.10	
Average monthly household income per capita	≤ 2000¥ /278.20$	291 (31.09)	3.60 ± 1.20	0.928
	2001 ~ 4000¥ /278.34$-553.40$	404 (43.16)	3.60 ± 1.20	
	4001 ~ 6000¥ /556.54$-834.60$	170 (18.16)	3.50 ± 1.10	
	6001 ~ 8000¥/834.74$-1112.80$	44 (4.70)	3.60 ± 1.30	
	> 8000¥ />1112.80$	27 (2.88)	3.50 ± 1.50	
Grade	Freshman	254 (27.14)	3.60 ± 1.10	0.037
	Sophomore	170 (18.16)	3.60 ± 1.20	
	Junior	250 (26.71)	3.60 ± 1.10	
	Senior	262 (28.00)	3.60 ± 1.30	
Father’s education level	Primary or low	333 (35.58)	3.60 ± 1.20	0.568
	Middle high school	405 (43.27)	3.60 ± 1.20	
	High or vocational school	142 (15.17)	3.50 ± 1.20	
	Undergraduate and above	56 (5.98)	3.70 ± 1.30	
Mothe’s education level	Primary or low	484 (51.71)	3.70 ± 1.20	0.024
	Middle high school	301 (32.16)	3.40 ± 1.20	
	High or vocational school	104 (11.11)	3.40 ± 1.30	
	Undergraduate and above	47 (5.02)	3.80 ± 1.10	
The preferred monthly remuneration	2000 ~ 4000 ¥/278.20$–553.40$	35 (3.74)	3.50 ± 0.90	0.729
	4001 ~ 6000 ¥/556.54$–834.60$	243 (25.96)	3.60 ± 1.20	
	6001 ~ 8000 ¥/834.74$–1112.80$	299 (31.94)	3.60 ± 1.20	
	8001 ~ 10,000 ¥/1112.80$–1391.00$	161 (17.20)	3.50 ± 1.20	
	> 10,000 ¥/>1391.00$	198 (21.15)	3.60 ± 1.30	

#### Correlations among variables

The Square root of Average Variance Extracted exceeded the correlation coefficients among constructs in all dimensions. The contents are summarized in Table 6.

#### Relationships between the theory of planned behaviour constructs

According to the TPB, significant correlations were observed among all dimensions. The contents are summarized in Table 6.

**Table 6:** Correlations among variables (*N* = 936)

	M ± SD (1–7)	Intention	Attitude	Subjective Norm	Perceived Behaviour Control	AVE	Root Square AVE	Composite Reliability
Intention	3.60 ± 1.20	1.00	0.43**	0.49**	0.63**	0.63	0.79	0.83
Attitude	4.60 ± 1.00	0.43**	1.00	0.52**	0.38**	0.57	0.75	0.79
Subjective Norm	4.20 ± 0.90	0.49**	0.52**	1.00	0.45**	0.65	0.81	0.85
Perceived Behaviour Control	3.70 ± 1.10	0.63**	0.38**	0.45**	1.00	0.62	0.79	0.83

**P < 0.001

#### Structural equation modeling of intentions of self-employment among Chinese undergraduate nursing students

**Fig. 2:**
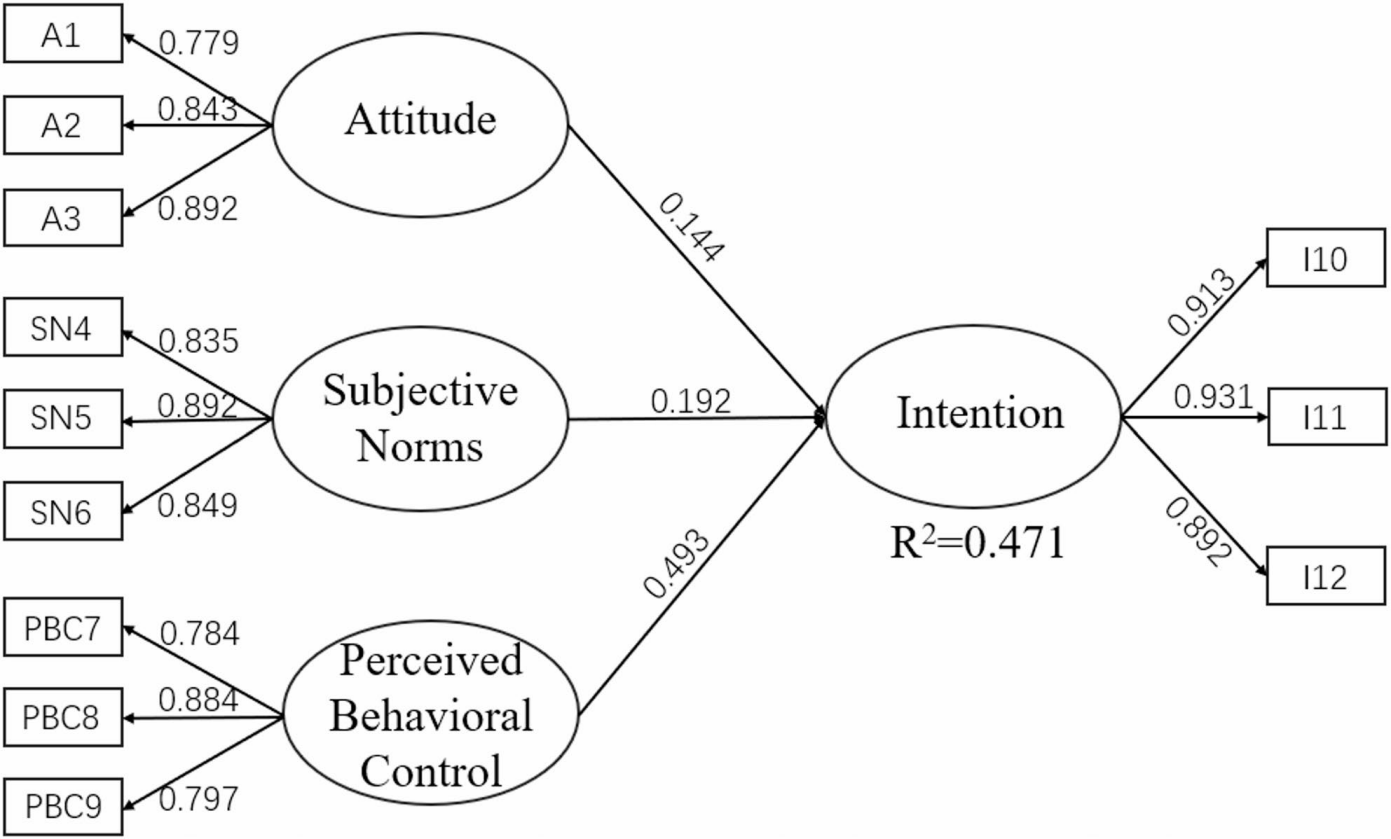
Significant path diagram of the model

The intention in this study had an R^2^ value of 0.47, suggesting that the model had a moderate level of explanatory power. Structural equation model analysis revealed that Attitude, Subjective Norm, and Perceived Behaviour Control had a significant positive effect on Intention. The contents are summarized in Fig. 2 and Table 7.

**Table 7:** Attitude, subjective norm and perceived behaviour control have positive effects on intention

Paths	Standardized coefficients (β)	Standard deviation	t	*p*
A → Intention	0.14	0.03	4.90	< 0.001
SN → Intention	0.19	0.03	5.89	< 0.001
PBC→ Intention	0.49	0.03	17.13	< 0.001

## Discussion

In this study, the English version of the PSES was translated into Chinese through cross-cultural adaptation. The Chinese version of the PSES has good reliability and validity and is suitable for the investigation of intentions and influencing factors of self-employment among Chinese undergraduate nursing students. Currently, the self-employment intention of Chinese undergraduate nursing students is at a moderate level. Attitudes, subjective norms and perceived behaviour control as influences on the intention of self-employment among Chinese undergraduate nursing students.

Intention serves as motivation to engage in a behavior and is viewed as the closest precursor to that behavior [19]. In the study, 47.10% of the intentions of self-employment among Chinese undergraduate nursing students was explained. This is a relatively moderate explanatory power compared to 55% from Bulfone’s study [12] on the predictor’s factors of the nursing students’ intention to be self-employed, 76.00% from Lim’s study [37] on the influencing factors of entrepreneurial intention among nursing students, 39.50% from Ha and Jung’s study on the influencing factors of entrepreneurial intention among college students [38]. Self-employment intention is the best predictor of self-employment behavior [39]. The study found that the average score in the intention dimension was consistent with the findings of Bulfone’s study [12], indicating that the intention of self-employment of nursing students was not clearly expressed. The theory of planned behavior suggests that intention is positively affected by attitudes, subjective norm, and perceived behaviour control. Therefore, it is necessary to take appropriate policy interventions to improve attitudes, subjective norms and perceived behaviour control, so as to increase the intention of self-employment among nursing students.

Attitude is a personal evaluation of behavior, and attitude is an important predictor of the intention of self-employment [19]. The study found that nursing students’ attitudes towards self-employed nurses had a positive effect on their intentions of self-employment(β = 0.14). Previous Qadasi’s studies [40] have demonstrated that attitudes towards self-employment have a strong and direct effect on self-employment intentions, and that positive attitudes lead to positive intentions to become self-employed nurses. Consistent with the findings of Al-Qadasi [40], attitudes significantly influence students’ self-employment intentions. Research indicates that personal satisfaction, improved quality of life, and the need for independence are the four core factors measuring entrepreneurial attitudes [41]. The anticipated outcomes of entrepreneurship—such as autonomy, stress levels, financial performance, personal satisfaction, and quality of life—significantly affect an individual’s likelihood of choosing entrepreneurship [42]. Existing research suggests that strengthening publicity and education through campus activities such as thematic education and dissemination of demonstration results can lead to improved positive attitudes [43]. Meanwhile, policy support should be provided to increase nursing students’ satisfaction and expectations of becoming self-employed nurses and to change self-employment attitudes by improving the registration and standardization system for self-employed nurses, thus ultimately promoting the intention of self-employment among nursing students [44].

Subjective norm are pressures from family or friends, society, and so forth to perform or not to perform behaviors and are key influences on the intention of self-employment [19]. Studies have shown that subjective norms have a positive effect on intention (β = 0.19), consistent with the findings of Mirjana et al. and Yang [45, 46]. Individuals’ entrepreneurial intentions correlate positively with subjective norms imposed by the external environment. Wall’s research [47] demonstrates that the level of acceptance of self-employed nurses by family members, coworkers, the public, and the government affects the intention to become a self-employed nurse, and it was noted that there is a traditional career culture in the society and that many citizens and families prefer stable jobs where they are recruited by the government and are not interested in jobs with financial risks. The intention rises with higher levels of social support. Thiessen [5] proposed that it is necessary to improve legal procedures and regulatory frameworks by expanding insurance coverage and lowering taxes as a way to enhance the social welfare of self-employed nurses and the recognition of self-employed nurses in order to promote the goals and operations of self-employed nurses, thereby increasing the intention of self-employment among nursing students [5, 44].

Perceived behaviour control relates to how easy or hard an individual believes it is to execute the behavior and also is an important factor influencing self-employment intentions [19]. The study’s findings indicate that perceived behaviour control positively affects intention (β = 0.49), consistent with the findings of Hossain et al. and Nguyen [48, 49]. Qadasi’s research [40] shows that in terms of self-employment, perceived behaviour control serves as self-employment self-efficacy, and entrepreneurship education can influence self-employment intentions by enhancing self-efficacy. Therefore, there is a necessity for the educational sector to emphasize nursing entrepreneurship education and to assist universities and colleges to establish relevant courses [50]. Placing emphasis on the College Students’ Innovative Entrepreneurial Training Plan Program for college students to provide students with the necessary knowledge and skills in order to develop nurses’ job competencies and core competencies. Moreover, policies such as clear guidelines for self-employment practice and competency assessment criteria should be enacted. These measures enable perceived behaviour control to be enhanced, which contributes to increasing the intention of self-employment among nursing students.

## Limitations

This study has several limitations that should be acknowledged. First, selection bias may have influenced the results due to the use of a convenience sampling method. This approach limits the generalizability of the findings, as the sample may not fully represent the broader population of nursing students in China. Furthermore, the relatively low response rate suggests that some nursing students who were not interested in self-employment may have opted not to participate, introducing self-selection bias. This could result in findings that are less comprehensive and potentially skewed toward students with stronger interest in self-employment. What’s more, the use of self-administered questionnaires is potentially subject to biases such as social desirability bias, participants may respond in a manner they perceive as socially acceptable rather than reflecting their genuine perspectives. Second, the study focused exclusively on undergraduate nursing students in Yunnan Province. This narrow sample selection limits the ability to generalize the findings to other groups, such as junior college nursing students or those from other regions in China. Additionally, the study did not explore the reliability and validity of the Chinese version of the PSES in the context of junior college nursing students, which is an important consideration for future research. Finally, as a cross-sectional study, this research is limited in its ability to establish causal relationships between variables. Future studies should consider longitudinal designs or larger, more diverse samples to better understand the dynamics of self-employment intentions and their influencing factors among nursing students in China.

## Conclusion

This study has identified the Chinese version of the PSES has good reliability and validity and is suitable for Chinese undergraduate nursing students. Attitudes, subjective norms, and perceived behaviour control were influential factors intention of self-employment among Chinese undergraduate nursing students, showing positive correlations. Therefore, urgent measures should be taken to enhance nursing students’ self-employment intentions by improving their attitudes, subjective norms, and perceived behaviour control toward self-employment. These measures include refining registration and regulatory frameworks, strengthening social welfare provisions for individual practice nurses, enhancing awareness and educational initiatives, and emphasizing the cultivation of innovation and entrepreneurial skills within the nursing profession.

## Supplementary Information

Below is the link to the electronic supplementary material.

Supplementary Material 1

Supplementary Material 2

## Data Availability

The datasets that were created during the course of the present study, as well as those that were analyzed during this study, can be obtained from the corresponding author. This is provided that the request made is reasonable.

## Abbreviations

PSES: The Planned Self-Employment Scale
I-CVI: Item-content validity index
S-CVI/Ave: The average scale-level content validity index
CRNS: College of Registered Nurses of Saskatchewan
NHS: The National Health Service
NLCs: Nurse-led clinics
IELTS: The International English Language Testing System
PLS-SEM: Partial least squares structural equation modeling
EFA: Exploratory factor analysis
CFA: Confirmatory factor analysis
GFI: Goodness of fit index
IFI: Incremental fit index
CFI: Comparative fit index
NFI: Normed fit index
TLI: Tucker-Lewis index
AGFI: Adjusted goodness-of-fit index
AVE: Average variance extracted
CR: Construct reliability
SEM: Structural equation modeling
SD: Standard deviation
