# Supplementary material for: Psychometric testing of the theory of planned behavior–based self-employment intention scale and its application among Chinese undergraduate nursing students
Source: BMC Nurs. 2025 Nov 26;24:1514. doi: 10.1186/s12912-025-04172-9 (PMC12752143; doi:10.1186/s12912-025-04172-9)
Supplement: Supplementary file 1 — Supplementary Material 1 [file 12912_2025_4172_MOESM1_ESM.docx]

**Supplementary materials**

The English version of the Planned Self-Employment Scale

| Dimension | Item |
| --- | --- |
| **Attitude** | 1. Being a self-employed nurse is more advantageous than disadvantageous for me. |
|  | 2. A self-employed nursing career is interesting to me. |
|  | 3. Being a self-employed nurse is very satisfying for me |
| **Subjective Norm** | 4. Being a self-employed nurse is very satisfying for my relatives |
|  | 5. Being a self-employed nurse is very satisfying for my friends |
|  | 6. Being a self-employed nurse is very satisfying for my colleagues  and/or fellow students |
| **Perceived**  **Behaviour**  **Control** | 7. Being a self-employed nurse could be easy for me |
|  | 8. I am ready to be a self-employed nurse |
|  | 9. I know the necessary procedures to follow to become a self-  employed nurse |
| **Intention** | 10. My professional goal is to become a self-employed nurse |
|  | 11. I am determined to become a self-employed nurse in the future |
|  | 12. I have a strong intention to become a self-employed nurse |

PSES score (1-7): 1 = totally disagree, 2 = more disagree than agree, 3 = somewhat disagree, 4 = neither disagree nor agree, 5 = somewhat agree, 6 = more agree than disagree, and 7 = totally agree.

The English version of the Planned Self-Employment Scale was developed by Bulfone et al. in 2020, comprising four dimensions, each containing three items, resulting in a total of 12 items. These dimensions are attitude, subjective norm, perceived behavioral control, and intention. The scale uses a 7-point Likert scale, with responses ranging from ‘totally disagree = 1’ to ‘totally agree = 7.’ The total score ranges from 12 to 84, while the scores for each dimension range from 3 to 21. Higher scores on each dimension indicate a stronger self-employment intention, more positive attitudes, and greater perceived behaviour control, while lower scores on the subjective norm dimension reflect less social pressure regarding self-employment.

中文版护士自雇意向量表

| 维度 | 条目 |
| --- | --- |
| **态度** | 1.对我来说，成为一名自雇护士是利大于弊的。 |
|  | 2.我对自雇护士这个职业很感兴趣。 |
|  | 3.对我来说，成为一名自雇护士是满意的。 |
| **主观规范** | 4.亲戚对于我做自雇护士这一职业感到满意。 |
|  | 5.朋友对于我做自雇护士这一职业感到满意。 |
|  | 6.同事和同学对于我做自雇护士这一职业感到满意。 |
| **知觉行为规范** | 7.做一名自雇护士，对我来说并不难。 |
|  | 8.我已经准备好成为一名自雇护士。 |
|  | 9.我知道成为一名自雇护士所需要的步骤。 |
| **意向** | 10.我的职业目标就是成为一名自雇护士。 |
|  | 11.我立志将来成为一名自雇护士。 |
|  | 12.我对于成为一名自雇护士的意向是强烈的。 |

中文版PSES得分(1-7)：1=完全不同意；2=不同意；3=有点不同意；4=不确定；5=有点同意；6=同意；7=完全同意

中文版护士自雇意向量表包含四个维度，每个维度包含三个条目，因此总共有12个条目。这些维度分别是态度、主观规范、感知行为控制和意向。该量表采用7点李克特量表，回答范围从“完全不同意=1”到“完全同意=7”。总分范围在12到84之间，而每个维度的分数范围在3到21之间。每个维度上得分越高表示自雇意向更强、态度更积极以及感知行为控制更强，而主观规范维度得分越低，则反映出在自雇方面较少的社会压力。
