# Supplementary material for: Psychometric testing of the theory of planned behavior–based self-employment intention scale and its application among Chinese undergraduate nursing students
Source: BMC Nurs. 2025 Nov 26;24:1514. doi: 10.1186/s12912-025-04172-9 (PMC12752143; doi:10.1186/s12912-025-04172-9)
Supplement: Supplementary file 2 — Supplementary Material 2 [file 12912_2025_4172_MOESM2_ESM.docx]

**Expert Interview Guide for Face Validity**

Dear Expert,

Thank you for taking the time to participate in our research interview amidst your busy schedules. We are conducting a study on the translation and cultural adaptation of *the Planned Self-Employment Scale (PSES)* into Chinese. The primary objective of this interview is to invite you to comprehensively review this Chinese version of the scale and assess whether it appears to measure the concept of “Chinese undergraduate nursing students’ self-employment intention” in a reasonable and clear manner. Your initial impressions and overall perceptions are crucial to our work. The interview content will be used solely for academic purposes and treated with strict confidentiality. The session is expected to last approximately 10-15 minutes.

**1. Overall Impression and Relevance:**

(1) After a quick review of this Chinese version of the scale, what are your initial impressions? Does it generally seem like an appropriate tool for measuring “Chinese undergraduate nursing students’ self-employment intention”?

(2) Do you believe the scale’s title, “the Chinese Version of the Planned Self-Employment Scale,” accurately and clearly reflects the content it aims to measure?

**2. Understandability and Clarity:**

(1) Without extensive reflection, do you find the scale’s instructions (i.e., the guidance provided to respondents) to be clear and comprehensible? Would Chinese undergraduate nursing students be able to complete the scale smoothly based on these instructions?

(2) Overall, do you consider the language used in the scale to be fluent and natural? Are there any sentences that appear awkward or difficult to understand upon reading?

**3. Cultural Appropriateness:**

(1) From a face perspective, do you think the content of this scale aligns with the educational and career contexts of Chinese undergraduate nursing students? Are there any elements that seem “Westernized” or inconsistent with the local environment?

**4. Overall Evaluation and Suggestions:**

(1) Based on your brief review, do you identify any apparent issues with the scale at a “face” level?

(2) Do you have any additional comments or suggestions derived from your first impression?

Once again, we sincerely appreciate your valuable time and insights. Your feedback will greatly contribute to the refinement of this scale.

**Expert Interview Guide for Content Validity**

Dear Expert,

We sincerely appreciate your participation in this expert consultation regarding the content validity of “*the Chinese Version of the Planned Self-Employment Scale (PSES)*” This interview requires you to apply your professional knowledge and experience to conduct an in-depth evaluation of each specific item on the scale, including its linguistic expression, content relevance, and importance. Your detailed feedback is crucial for ensuring the scientific rigor of the scale. The interview is expected to take 20-30 minutes, and all your opinions will be treated with strict confidentiality.

**Part I: Overall Assessment**

1. To what extent does the Chinese Version of the Planned Self-Employment Scale comprehensively cover the core dimensions of the concept “Chinese undergraduate nursing students’ self-employment intention”? (For example, does it cover key aspects such as self-employment intention, attitudes toward self-employment, subjective norms, and perceived behaviour control?)

2. Are there any important elements you believe are missing from the scale? Conversely, are there any items you consider unnecessary or irrelevant?

**Part II: Item-by-Item Evaluation**

(Please review the scale while providing comments on each item regarding the following aspects)

**1. Language and Grammar:**

(1) Is the Chinese translation of this item accurate? Does it exhibit awkward literal translation or ambiguity?

(2) Is the vocabulary selection appropriate? Does it align with the linguistic habits and comprehension level of Chinese undergraduate nursing students?

(3) Is the grammar and sentence structure clear and unambiguous?

**2. Content Relevance:**

(1) How relevant is this item to the concept of “nursing students’ self-employment intention”?

(2) Does this item adequately measure the dimension it is intended to measure?

**3. Importance:**

(1) How important is this item for measuring nursing students’ self-employment intention?

**4. Clarity and Precision:**

(1) Is the wording of this item clear and specific? Could nursing students accurately understand its meaning?

(2) Do you perceive any potential for this item to be misunderstood? If so, in what aspects?

**5. Item Construction and Scoring:**

(1) Is the construction of this item (including its statement format) reasonable?

(2) Is the corresponding response scale (e.g., Likert scale) appropriate? Can it effectively differentiate between the intention levels of different nursing students?

**Part III: Comprehensive Recommendations**

1. Among all items, which do you consider most suitable/least suitable for measuring self-employment intention? Please explain why.

2. For items requiring modification, what specific revision suggestions or alternative wording would you propose?

3. From an overall content validity perspective, what final general comments or recommendations do you have for this scale?

Your meticulous and professional evaluation provides invaluable guidance for our revision process. We extend our deepest gratitude for your generous support.
